# Photochemistry in the strong coupling regime: A trajectory surface hopping scheme

**DOI:** 10.1002/jcc.26369

**Published:** 2020-07-01

**Authors:** Jacopo Fregoni, Stefano Corni, Maurizio Persico, Giovanni Granucci

**Affiliations:** ^1^ Dipartimento di Scienze Fisiche Informatiche e Matematiche University of Modena and Reggio Emilia Modena Italy; ^2^ Dipartimento di Scienze Chimiche University of Padova Padova Italy; ^3^ Dipartimento di Chimica e Chimica Industriale University of Pisa Pisa Italy

**Keywords:** nonadiabatic dynamics, photochemistry, polaritonic chemistry, strong coupling, surface hopping

## Abstract

The strong coupling regime between confined light and organic molecules turned out to be promising in modifying both the ground state and the excited states properties. Under this peculiar condition, the electronic states of the molecule are mixed with the quantum states of light. The dynamical processes occurring on such hybrid states undergo several modifications accordingly. Hence, the dynamical description of chemical reactivity in polaritonic systems needs to explicitly take into account the photon degrees of freedom and nonadiabatic events. With the aim of describing photochemical polaritonic processes, in the present work, we extend the direct trajectory surface hopping scheme to investigate photochemistry under strong coupling between light and matter.

## Graphical abstract

The molecular electronic potential energy landscape is modified by effect of cavity‐molecule strong coupling. In turn, this affects the motion of the nuclear wavepacket on the potential energy surfaces, resulting in potentially modified photochemical reactivity. In the current work, we present the quantum chemical theory aimed at the description of photochemical processes occurring when the electronic states hybridise with quantum light.

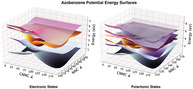

## 1 INTRODUCTION

The coherent interaction between light and matter in confined systems offers an alternative pathway to tailor optical and chemical properties of molecules. While the spectroscopy of atoms and molecules in resonant cavities is well established, the possibility to manipulate the molecular reactivity through quantum coupling with light has only recently been addressed. By devising microcavities^[^ ^1^, ^2^ ^]^ and nanocavities,^[^ ^3^, ^4^ ^]^ the experimental efforts^[^ ^5^, ^6^ ^]^ to bring molecules in the strong coupling regime down to the single molecule level have driven an increasing theoretical interest.^[^ ^7^, ^8^, ^9^, ^10^ ^]^ Yet, the modeling of such complex systems experiences limitations both theoretical and computational.

Understanding which approximations can hold for a correlated nuclear–photonic–electronic system is indeed challenging.^[^ ^11^, ^12^ ^]^ Even more, an important option is whether to couple the photonic degrees of freedom to the nuclear ones or to the electronic ones.^[^ ^8^, ^13^, ^14^ ^]^ Within the first approach, the photonic degrees of freedom are treated so as the nuclear ones, allowing to study the effect of the electron–nuclei–photon coupling on adiabatic potential energy surfaces. Such approach provides insightful tools of analysis for phenomena like Raman Scattering,^[^ ^15^, ^16^ ^]^ modified molecular properties,^[^ ^14^, ^17^ ^]^ and ground state reactivity.^[^ ^18^, ^19^, ^20^ ^]^ Instead, the second approach, in which electronic and photonic states are mixed, is suitable to describe the modified photochemical properties^[^ ^8^, ^21^ ^]^ and reactivity,^[^ ^22^, ^23^, ^24^ ^]^ provided that nonadiabatic couplings are taken into account.

A full quantum approach has been developed by Rubio's group in the DFT framework. The method is based on rewriting the DFT formulation in terms of a current density functional which allows to include the photonic degrees of freedom^[^ ^10^, ^25^ ^]^ (QEDFT). Later on, the same group reformulated the Born–Oppenheimer approximation to partially decouple the nuclear–photonic–electronic problem with the so‐called Cavity Born–Oppenheimer approximation.^[^ ^13^, ^14^ ^]^ Such works opened a way to a full ab‐initio investigation of strongly coupled light–matter systems,^[^ ^10^, ^17^, ^26^ ^]^ with successful applications in strong‐coupling modified properties of single and many molecules.

Aiming to investigate polaritonic photochemical reactions, the complexity of the system can quickly become cumbersome. The correct computation of excited states is mandatory, together with the treatment of the photonic degree of freedom.^[^ ^27^, ^28^, ^29^ ^]^ Further complexity to the problem is added by interfacing a propagation scheme for the nuclei^[^ ^30^ ^]^ and by accounting for environmental effects. In addition, the common problems encountered in photochemical simulations^[^ ^31^ ^]^ are directly transposed to the study of polaritonic photochemical reactions.

A pioneering conceptual display of novel photochemical events in the strong coupling regime is offered by the works of Mukamel and collaborators^[^ ^7^, ^32^, ^33^ ^]^ and Feist and collaborators^[^ ^8^, ^22^, ^23^ ^]^ on model molecules. Such works collect a plethora of insights for a novel chemical reactivity ranging from single‐molecule strong coupling to collective strong coupling effects. We have also recently shown how moving beyond model treatments to investigate polaritonic chemistry can also reveal noteworthy effects like enhanced photoisomerization quantum yields.^[^ ^34^ ^]^

To simulate mixed light–molecule systems, a toolbox of strong‐coupling techniques for photochemistry has been developed in the last three years.^[^ ^24^, ^35^, ^36^ ^]^ Among them, we mention the multiscale MD approach devised by Groenhof and collaborators, which allows to investigate the collective polariton behavior in biological environments through a QM/MM approach.^[^ ^35^, ^37^, ^38^ ^]^ For events occurring in small ensembles of realistic molecules in cavities on a shorter timescale, the extension of the MCTDH technique to polaritonic systems^[^ ^36^, ^39^ ^]^ is also remarkable. In recent works,^[^ ^24^, ^34^ ^]^ we showed how the Surface Hopping scheme in a semiempirical framework can be used to describe the photochemistry of molecules in a strong coupling environment with a high level of realism. A similar Surface Hopping scheme has been used by Tretiak et al.,^[^ ^40^ ^]^ which studied the stilbene photoisomerization under strong coupling, employing a single reference quantum chemical approach for the electronic states. Comparatively, in our scheme, we also include cavity losses, and our semiempirical multireference FOMO‐CI scheme allows for a qualitatively correct description of potential energy surfaces and couplings, which is also quantitatively accurate since we reparametrize the semiempirical Hamiltonian.

In the present contribution, we show in detail the theoretical approximations and the implementation techniques of our approach to polaritonic chemistry. To this aim, we show first how polaritonic states are built on top of the semiempirical FOMO‐CI^[^ ^41^, ^42^, ^43^, ^44^ ^]^ technique for the computation of electronic states. Then, we derive the analytical gradients for the strong coupling contribution to the CI energy via the Z‐vector^[^ ^45^, ^46^ ^]^ algorithm. We also discuss the interface with the on‐the‐fly Direct Trajectory Surface Hopping (DTSH), with emphasis on the method we have adopted to include the effect of cavity losses on the dynamics.

We want to stress that the hereby presented gradients and Surface Hopping interface have a general applicability to multiconfigurational wavefunction methods. The choice of a semiempirical approach to solve the electronic problem resides in the good compromise between efficiency and accuracy.^[^ ^31^ ^]^ We also mention that such approach has been successfully applied to deal with the molecular complexity of polaritons when all the degrees of freedom are taken into account,^[^ ^24^ ^]^ and also in the presence of an environment^[^ ^34^ ^]^ inspired to a realistic setup.^[^ ^5^ ^]^ We also stress that, while our method carries the potential to treat a few chromophores, the study of a large ensemble of molecular systems is beyond the aim of the present work.

## 2 METHODOLOGY

### 2.1 Polaritonic wavefunction in a semiclassical framework

To build polaritonic states, we consider a generalized correlated photon–electron–nuclear system:

(1) $${\hat{H}}_{\mathrm{tot}}={\hat{T}}_{e}+{\hat{T}}_{n}+{\hat{T}}_{\mathrm{ph}}+{\hat{W}}_{e,e}+{\hat{W}}_{e,n}+{\hat{W}}_{n,n}+{\hat{W}}_{e,\mathrm{ph}}+{\hat{W}}_{\mathrm{ph},n}+{\hat{W}}_{\mathrm{ph},n,e}$$

where the electronic degrees of freedom are described by the *e* subscript (***r*** coordinates), the nuclear ones by the *n* subscript (***R*** coordinates), and the photon one by the *ph* subscript (***q*** coordinates). The total wavefunction of the correlated electron–photon–nuclei system is Ψ(***r***, ***R***, ***q***). Two approaches to approximate the eigenstates and the time‐evolution of strongly coupled systems have been applied so far: the first is to embed the photon degrees of freedom into the nuclear wavefunction^[^ ^14^ ^]^ while the second is to embed the photon into the electronic wavefunction.^[^ ^8^ ^]^

Such two different approaches provide different insight on two classes of processes. In fact, the molecular properties and the dynamics in the Cavity Born–Oppenheimer approximation^[^ ^13^, ^17^ ^]^ are optimally described by incorporating the photon in the nuclear wavefunction (Ψ_*n* + *ph*, *e*_). Instead, the processes involving nuclear dynamics on polaritonic states, that is, photochemical processes, are accurately described by considering hybrid electron–photon states (polaritons, Ψ_*n*, *e* + *ph*_).^[^ ^7^, ^24^, ^32^, ^34^, ^47^ ^]^ The Born–Huang factorizations of the wavefunction in these cases respectively correspond to:

(2) $$\Psi_{n+\mathrm{ph},e}\left(r,q,R,t\right)=\sum_{k}\;\chi_{k}\left(R,q,t\right)\;\phi_{k}^{\mathrm{el}}\;\left(r;q,R\right),$$

(3) $$\Psi_{n,e+\mathrm{ph}}\left(r,q,R,t\right)=\sum_{k}\;\chi_{k}\;\left(R,t\right)\;\phi_{k}^{e+\mathrm{ph}}\;\left(r,q;R\right).$$

Equation (2) represents the case where the photon degrees of freedom are considered slow. Hence, they are treated alike to the nuclear degrees of freedom in the Cavity Born–Oppenheimer framework.^[^ ^13^, ^17^ ^]^ Based on this assumption, the purely electronic wavefunction and the related electronic potential energy surfaces show a parametric dependence on both the nuclear and photonic coordinates. This framework explicitly requires to compute the quantum nuclear wavefunction to include the photon effects, hence it is not properly interfaced with semiclassical methods developed treating the whole nuclear dynamics as classical.

In the factorization presented in Equation (3), the photonic degrees of freedom are considered fast and possibly resonant with optical transitions. Within this framework, the parametric dependence of the mixed electronic‐photonic wavefunction with respect to the nuclear degrees of freedom allows to describe the time evolution of a polaritonic wavefunction with semiclassical trajectory‐based methods. In that case, the nuclei are moving according to a classical trajectory ***R***(*t*), and the polaritonic nonadiabatic couplings can be included as for the purely electronic case. In the semiclassical case, we define by analogy with Equation (3) the polaritonic wavefunction:

(4) $$|\Psi_{\mathrm{pol}}\left(r,q,R\left(t\right),t\right)〉=\sum_{A}C_{A}\left(t\right)|A\left(r,q;R\right)〉,$$

where ∣*A*〉 are the semiclassical analogous of the polaritonic states $\phi_{k}^{\mathrm{ph}+e}$ of Equation (3). We choose ∣*A*〉 to label such states to directly refer to their adiabatic behavior, as they are the eigenstates of the polaritonic Hamiltonian:

(5) $${\hat{H}}_{\mathrm{pol}}|A〉=E_{A}|A〉,$$

with

(6) $${\hat{H}}_{\mathrm{pol}}={\hat{H}}_{\mathrm{el}}+{\hat{H}}_{\mathrm{ph}}+{\hat{H}}_{\mathrm{int}}.$$

Here, ${\hat{H}}_{\mathrm{el}}$ is the standard electronic Hamiltonian and ${\hat{H}}_{\mathrm{ph}}$ is the Hamiltonian of the quantized electromagnetic field (we consider here a single mode for the field),

(7) $${\hat{H}}_{\mathrm{ph}}=\hbar \omega_{\mathrm{ph}}\left({\hat{b}}^{†}\hat{b}+\frac{1}{2}\right)$$

where ${\hat{b}}^{†}$, $\hat{b}$ are the creation and annihilation operators for the electromagnetic field. In principle, the approach considered in this work could be extended in a straightforward way to consider several cavity modes. However, it is uncommon that many modes can reach a coupling strength large enough to require a strong coupling treatment, not to mention that they may also be well separated in energy. As interaction Hamiltonian *H*_*int*_, we take the dipolar light–matter interaction in the Coulomb gauge and long wavelength approximation:

(8) $${\hat{H}}_{\mathrm{int}}={ℰ}_{1\mathrm{ph}}\lambda \;\cdot \;{\hat{\mu }}_{\mathrm{tr}}\left({\hat{b}}^{†}+\hat{b}\right).$$

In the light–matter interaction, we refer to ℰ_1*ph*_ as the 1‐photon field strength, with the electromagnetic field polarization ***λ***. ${\hat{\mu }}_{\mathrm{tr}}$ is the electronic transition dipole moment between the electronic states. Notice that ${\hat{H}}_{\mathrm{pol}}$ is parametrically dependent on the nuclear coordinates through ${\hat{H}}_{\mathrm{el}}$ and ${\hat{H}}_{\mathrm{int}}$. As we have numerically verified in previous works,^[^ ^24^, ^34^ ^]^ for the case of strong coupling with optical frequencies, it is enough to restrain to the transition dipole operator. In the next section we focus term‐by‐term on the two individual subcomponents of the polaritonic Hamiltonian, namely ${\hat{H}}_{\mathrm{el}}$ and ${\hat{H}}_{\mathrm{ph}}$.

### 2.2 FOMO‐CI wavefunction and uncoupled states

The method for the computation of electronic states, that is, the eigenstates of ${\hat{H}}_{\mathrm{el}},$ is based on floating occupation of molecular orbitals (FOMO).^[^ ^42^, ^43^ ^]^ This variant relies on the optimization of a single determinant wavefunction with fractional variational occupation of the molecular orbitals through a self‐consistent field calculation (SCF). The single‐determinant SCF calculation is formally closed‐shell. Here, the energy of the *i*‐th orbital (*φ*_*i*_) is the Fock eigenvalue *ε*_*i*_ corresponding to that orbital, while the occupation number *O*_*i*_ of *φ*_*i*_ is obtained integrating a function *f*_*i*_(*ε*) normally distributed along the energy axis around *ε*_*i*_:

(9) $$\hat{F}\varphi_{i}=\varepsilon_{i}\varphi_{i},$$

(10) $$O_{i}=\int_{-\infty }^{\varepsilon_{F}}f_{i}\left(\varepsilon \right)\mathrm{d\varepsilon}=\int_{-\infty }^{\varepsilon_{F}}\frac{\sqrt{2}}{\sqrt{\pi }\sigma }e^{-\frac{{\left(\varepsilon -\varepsilon_{i}\right)}^{2}}{2\sigma^{2}}}\mathrm{d\varepsilon}.$$

Here, *σ* is an arbitrary parameter and the Fermi energy *ε*_*F*_ is determined by imposing that the sum of the orbital occupation numbers *O*_*i*_ equals the total number of electrons. The Fock operator $\hat{F}$ is obtained from the density (orbitals are considered as real functions in the present work)

(11) $$\rho \left(\vec{r}\right)=\sum_{i}O_{i}\varphi_{i}^{2}\left(\vec{r}\right).$$

Through this procedure, the lower virtual orbitals can be populated without resorting to a MCSCF optimization of the wavefunction, allowing to smoothly adapt the orbitals to the internal coordinate's variations with just a single determinant. The electronic wavefunctions are obtained performing a CI calculation on top of the FOMO‐SCF, resulting in a multiconfigurational FOMO‐CI. This approach can be taken as a replacement of the more accurate but much more complex CASSCF procedure.

As electronic Hamiltonian ${\hat{H}}_{\mathrm{el}}$, we consider a semiempirical Hamiltonian, as this allows to treat relatively large chromophores, including all the degrees of freedom in the simulation of polaritonic photochemistry, for timescales up to several picoseconds. In particular, for our test case we used a reparametrized version of the AM1 semiempirical Hamiltonian.^[^ ^41^ ^]^ Notice that the standard semiempirical parameters are normally determined to reproduce ground state properties, with a SCF wavefunction. Therefore, to deal with excited states, a reparametrization is often mandatory, as what has been done in Reference [41].

As we adopt a CI‐type wavefunction, the (approximated) eigenstates ∣*n*〉 of ${\hat{H}}_{\mathrm{el}}$ and the corresponding eigenenergies *U*_*n*_ are obtained by diagonalizing the electronic Hamiltonian matrix:

(12) $${\hat{H}}_{\mathrm{el}}|n〉=U_{n}|n〉,$$

on the basis of a chosen set of *N*_*CI*_ Slater's determinants {Φ}, so that

(13) $$|n〉=\sum_{K}^{N_{\mathrm{CI}}}C_{K,n}|\Phi_{K}〉$$

Similarly to the electronic states, the photon states are the eigenstates ∣*p*〉 of ${\hat{H}}_{\mathrm{ph}}$:

(14) $${\hat{H}}_{\mathrm{ph}}|p〉=\hbar \omega_{\mathrm{ph}}\left(p+\frac{1}{2}\right)|p〉.$$

The meaning of *p* is a photon occupation state number, for the single electromagnetic mode of frequency *ω*_*ph*_ considered here.

The product states between the electronic and photonic eigenstates ∣*n*, *p*〉 are then the eigenstates of the light–matter noninteracting Hamiltonian ${\hat{H}}_{\mathrm{el}}+{\hat{H}}_{\mathrm{ph}}$. We shall address to them as uncoupled states through all the present work. Such set of uncoupled states ∣*n*, *p*〉 are the polaritonic equivalent of, for example, the spin‐diabatic states for the purely electronic case with spin‐orbit coupling.^[^ ^48^ ^]^

### 2.3 Polaritonic states evolution and energies

The time evolution of the wavefunction is performed in terms of the polaritonic adiabatic states ∣*A*〉, which are obtained by diagonalization of the matrix of ${\hat{H}}_{\mathrm{pol}}$ (Equation (6)) on a selected subspace of *N* × (*p*_*max*_ + 1) uncoupled states {*n*, *p*}, where *N* ≤ *N*_*CI*_ is the number (usually small) of electronic states considered, and *p*_*max*_ is the maximum value of the photon occupation number. The set of adiabatic states ∣*A*〉 is used to perform the time evolution as the surface hopping approach is representation‐dependent, and usually performs better in the adiabatic basis. However, the set of uncoupled states ∣*n*, *p*〉 is still useful, mainly in order to ease the interpretation of the results. Within the described framework, the polaritonic wavefunction evolves according to the “polaritonic TDSE” $i\hbar {\dot{\Psi }}_{\mathrm{pol}}={\hat{H}}_{\mathrm{pol}}\Psi_{\mathrm{pol}}$, which gives

(15) $${\dot{C}}_{A}\left(t\right)=-\sum_{B}\left(\frac{i}{\hbar }H_{\mathrm{AB}}+〈A\left|\frac{d}{\mathrm{dt}}\right|B〉\right)C_{B}\left(t\right)$$

(16) $$=-\sum_{B}\left(\frac{i}{\hbar }H_{\mathrm{AB}}+G_{\mathrm{AB}}\cdot \dot{R}\left(t\right)\right)C_{B}\left(t\right),$$

where $H_{\mathrm{AB}}=〈A\left|{\hat{H}}_{\mathrm{pol}}\right|B〉$ and ***G***_*AB*_ is the derivative coupling vector between the polaritonic states ∣*A*〉 and ∣*B*〉, namely

(17) $$G_{\mathrm{AB}}=〈A\left|{\hat{\text{∇}}}_{R}\right|B〉.$$

According to Equation (13), a polaritonic state can be written as

(18) $$|A〉=\sum_{n=1}^{N}\sum_{p=0}^{p_{\max}}D_{n,p}^{A}|n,p〉=\sum_{n,p}D_{n,p}^{A}\sum_{K}^{N_{\mathrm{CI}}}C_{K,n}|\Phi_{K},p〉,$$

and its energy is

(19) $$E_{\mathrm{pol}}^{A}=E_{\mathrm{el}}^{A}+E_{\mathrm{ph}}^{A}+E_{\mathrm{int}}^{A},$$

where the contribution of the uncoupled part can be extracted by exploiting Equations (14) and (18), resulting in:

(20) $$E_{\mathrm{el}}^{A}=\sum_{n}U_{n}\sum_{p}{\left|D_{n,p}^{A}\right|}^{2}$$

(21) $$E_{\mathrm{ph}}^{A}=\hbar \omega_{\mathrm{ph}}\left(\sum_{n,p}p{\left|D_{n,p}^{A}\right|}^{2}+\frac{1}{2}\right).$$

The interaction term $E_{\mathrm{int}}^{A}$ is given by

(22) $$E_{\mathrm{int}}^{A}={ℰ}_{1\mathrm{ph}}\sum_{n\neq m}〈m|\lambda \cdot \hat{\mu }\left(R\right)|n〉D\left(A|m,n\right)$$

where we used the shorthand

(23) $$D\left(A|m,n\right)=\sum_{p=0}^{p_{\max}-1}\sqrt{p+1}\left(D_{n,p}^{A}D_{m,p+1}^{A}+D_{n,p+1}^{A}D_{m,p}^{A}\right).$$

Notice that *D*(*A* ∣ *m*, *n*) = *D*(*A* ∣ *n*, *m*).

When *m* < *n* the process described is the molecule exchanging the photon of frequency *ω*_*ph*_ with the cavity. The rate of such exchange is the Rabi splitting (Jaynes‐Cummings Hamiltonian).^[^ ^49^, ^50^ ^]^ In this regime, the emission rate and efficiency is greatly enhanced through the Purcell effect^[^ ^47^, ^51^ ^]^ and the energy is coherently exchanged between matter and cavity. Such energy contribution is the Rabi splitting. Instead, when *m* > *n*, the so‐called counter rotating terms account for the simultaneous creation/annihilation of two off‐resonant excitations within the cavity. Such terms become non‐negligible, together with the dipolar self‐energy of the molecule, in the ultrastrong coupling regime.^[^ ^11^, ^12^ ^]^

From now on, we will use *i*, *j*, … to label CI‐active molecular orbitals (MO) and *a*, *b* for any kind of MO. A more appealing expression, from the computational point of view, of the interaction energy $E_{\mathrm{int}}^{A}$ is obtained by using the spinless electronic density matrix, suitably modified, of the polaritonic state ∣*A*〉 considered. In particular, we have

(24) $$E_{\mathrm{int}}^{A}={ℰ}_{1\mathrm{ph}}\sum_{\mathrm{ij}}\rho_{\mathrm{ij}}^{\mathrm{int}}\left(A\right)\mu_{\mathrm{ij}},$$

where $\mu_{\mathrm{ij}}=〈i|\lambda \cdot \hat{\mu }|j〉$ and

(25) $$\rho_{\mathrm{ij}}^{\mathrm{int}}\left(A\right)=\sum_{n\neq m}D\left(A|m,n\right)\Delta_{\mathrm{ij}}^{\mathrm{el}}\left(m,n\right).$$

$\Delta_{\mathrm{ij}}^{\mathrm{el}}\left(m,n\right)$ is the spinless transition density matrix between the electronic states *m* and *n*, expanded on the molecular orbital basis. The action of the bosonic creators and annihilators of Equation (8) is embedded into the *D*(*A* ∣ *m*, *n*) coefficients. Therefore, $\Delta_{\mathrm{ij}}^{\mathrm{el}}\left(m,n\right)$ is purely electronic:

(26) $$\Delta_{\mathrm{ij}}^{\mathrm{el}}\left(m,n\right)=〈m|{\hat{a}}_{i}^{†}{\hat{a}}_{j}|n〉=\sum_{I,J}C_{I,m}〈\Phi_{I}|{\hat{a}}_{i}^{†}{\hat{a}}_{j}|\Phi_{J}〉C_{J,n}$$

Within our method, we are able to compute the Polaritonic Potential Energy Surfaces (PoPESs) up to an arbitrary occupation number of the photonic mode involved in strong coupling.

We shall now briefly discuss the dependence of the PoPESs on the molecular transition dipole moments. Upon diagonalization, a crossing of the uncoupled states PES is converted to a polaritonic avoided crossing. The magnitude of the splitting (Rabi splitting) is proportional to the transition dipole moment between the crossing states, potentially reaching zero for vanishing transition dipole moments (polaritonic conical intersection). The strong dependence of the Rabi splitting on the transition dipole moment also embodies a strong dependence on the nuclear geometry at which the crossing between uncoupled states occurs, as the transition dipole moments variation with nuclear geometry may be large. Note that the orientation of the molecule here plays the same role as the internal coordinates, because it affects the projection of the transition dipole on the field polarization vector.

**FIGURE 1:**
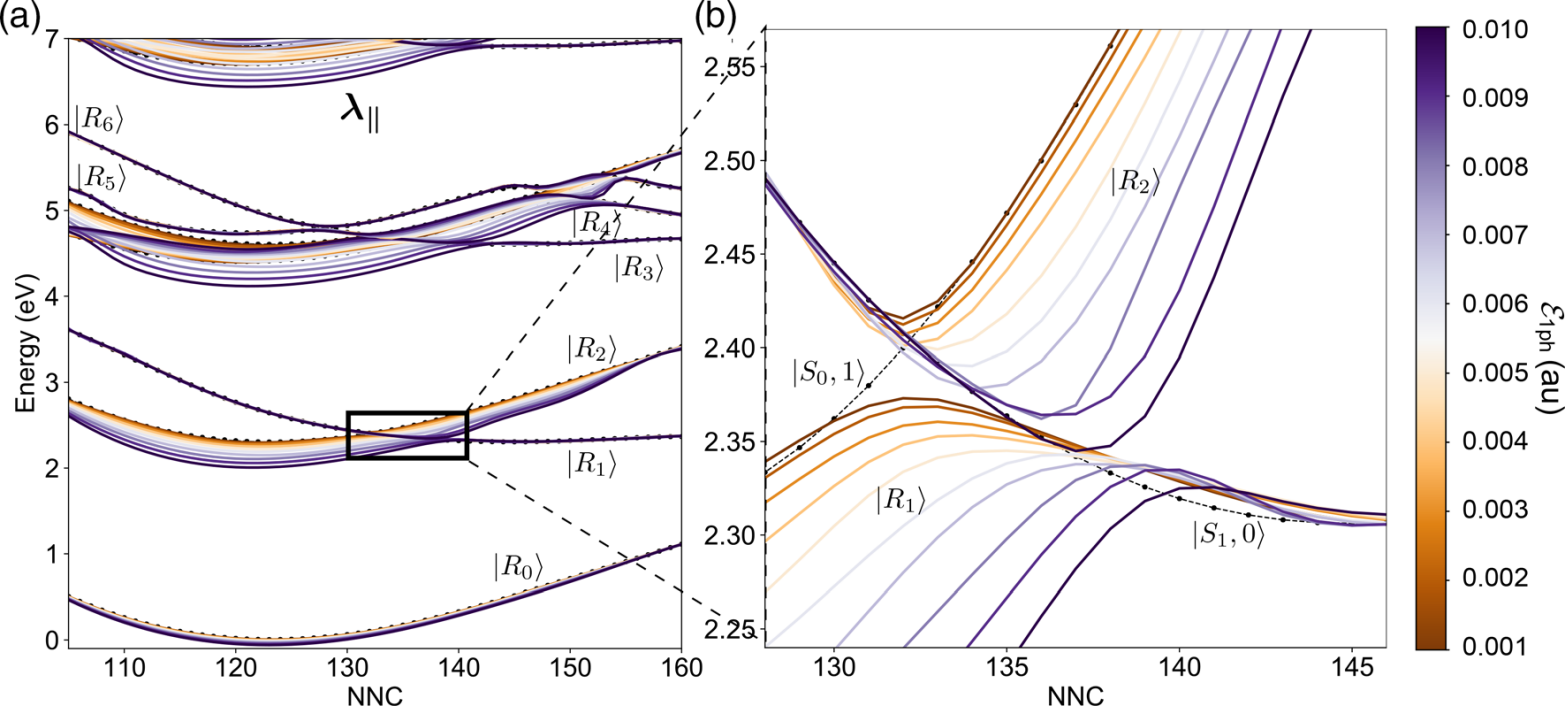
Polaritonic crossing seams for weak to ultrastrong values of ℰ_1*ph*_. (a) Polaritonic PESs along the NNC coordinate with CNNC fixed at 175°, relaxing all the other degrees of freedom and including multiple states and counter‐rotating terms. The field polarization ***λ***_∥_ is taken parallel to the longitudinal axis of the molecule. Although values of ℰ_1*ph*_ > 0.005 au (<7 nm^3^ mode volume) can actually be reached through single‐atom hotspots,^[^ ^54^, ^55^ ^]^ (b) a drastic effect on the PESs shape is observed also for intermediate values of ℰ_1*ph*_, ranging from 0.002 au (∼46 nm^3^ mode volume) to 0.004 au (∼12 nm^3^ mode volume), resulting in the seam shifting up to 3–4° along the NNC coordinate. In both panels, the dotted‐dashed lines label purely electronic states (no strong coupling) [Color figure can be viewed at wileyonlinelibrary.com]

While the polaritonic conical intersection and avoided crossings have been reported in previous works,^[^ ^8^, ^24^, ^39^, ^52^, ^53^ ^]^ here we stress that they are an easy‐to‐predict feature only when limited to two level strong coupling models, that is, Jaynes‐Cummings like. Two‐level models imply a linear dependence of the Rabi splitting on the coupling constant ℰ_1*ph*_. As the number of electronic states is extended by including upper states (Figure 1a), the interaction between the polaritons originating the avoided crossing or conical intersections becomes more involved. This behavior is due to the interaction between the uncoupled states not directly crossing, originated by the counter‐rotating terms in the Hamiltonian. The sum of such interactions deeply affects the polaritonic energy landscape by modifying both the splitting and the crossing geometry, as shown in Figure 1a,b for the azobenzene molecule.

We stick to azobenzene as a test case, since the phenomenology of polaritonic photochemistry has been investigated in recent works.^[^ ^24^, ^34^ ^]^ In the present work, instead, we focus on discussing the change of shape of the polaritonic avoided crossing regions, computed along NNC for different values of ℰ_1*ph*_. For mode volumes smaller than 20 nm^3^ (ℰ_1*ph*_ > 0.003), the polaritonic crossing seam gets displaced to up to 8° along the NNC coordinate while the Rabi splitting is not much affected, as shown in Figure 1. The curves here are computed within a model space of uncoupled states composed by 5 electronic states and photon occupation number ranging from 0 to 3. The polaritonic state energies are computed along the symmetric NNC bending angles with fixed CNNC (180°) and optimizing all the other degrees of freedom for the ground state energy, resulting in a *C*_2_ symmetry. The photon frequency is set at 2.30 eV and the polarization of the field is oriented along the longitudinal axis of the molecule. Here, the high transition dipole moment between the state *S*_0_ and the *S*_2_, *S*_3_, and *S*_4_ states manifold is instrumental in modifying the avoided ∣*S*_0_, 1〉,∣*S*_1_, 0〉 crossing landscape by effect of the interaction between the state ∣*S*_0_, 1〉 and the ∣*S*_2_, 0〉, ∣*S*_3_, 0〉, and ∣*S*_4_, 0〉 manifold.

We examine the whole range of ℰ_1*ph*_ going from 0.002 au (corresponding to a mode volume of ∼40 nm^3^) to ℰ_1*ph*_ = 0.010 au (∼1.6 nm^3^). While a mode volume of 40 nm^3^ is in line with typical nanocavities,^[^ ^5^ ^]^ the extreme limit of ∼1 nm^3^ has been accessed experimentally via single‐atom hotspots.^[^ ^54^, ^55^ ^]^ In all the conditions examined in this work, the mode volume is enough to fully embed the molecule (the molecular volume being ∼0.25 nm^3^). A few works pioneer the interaction beyond the dipolar approximation for small mode volumes for TERS experiments,^[^ ^56^, ^57^ ^]^ but not in connection with polaritonic photochemistry. More practically, it is not clear at which volumes and in which conditions the dipolar approximation ceases to be valid in the framework of dynamical processes. Moving beyond the dipolar treatment for polaritonic photochemistry carries the promise to reveal new effects for strong coupling at submolecular level. However, in the present case, we limit ourselves to the dipolar approximation for the whole range of mode volumes investigated.

**FIGURE 2:**
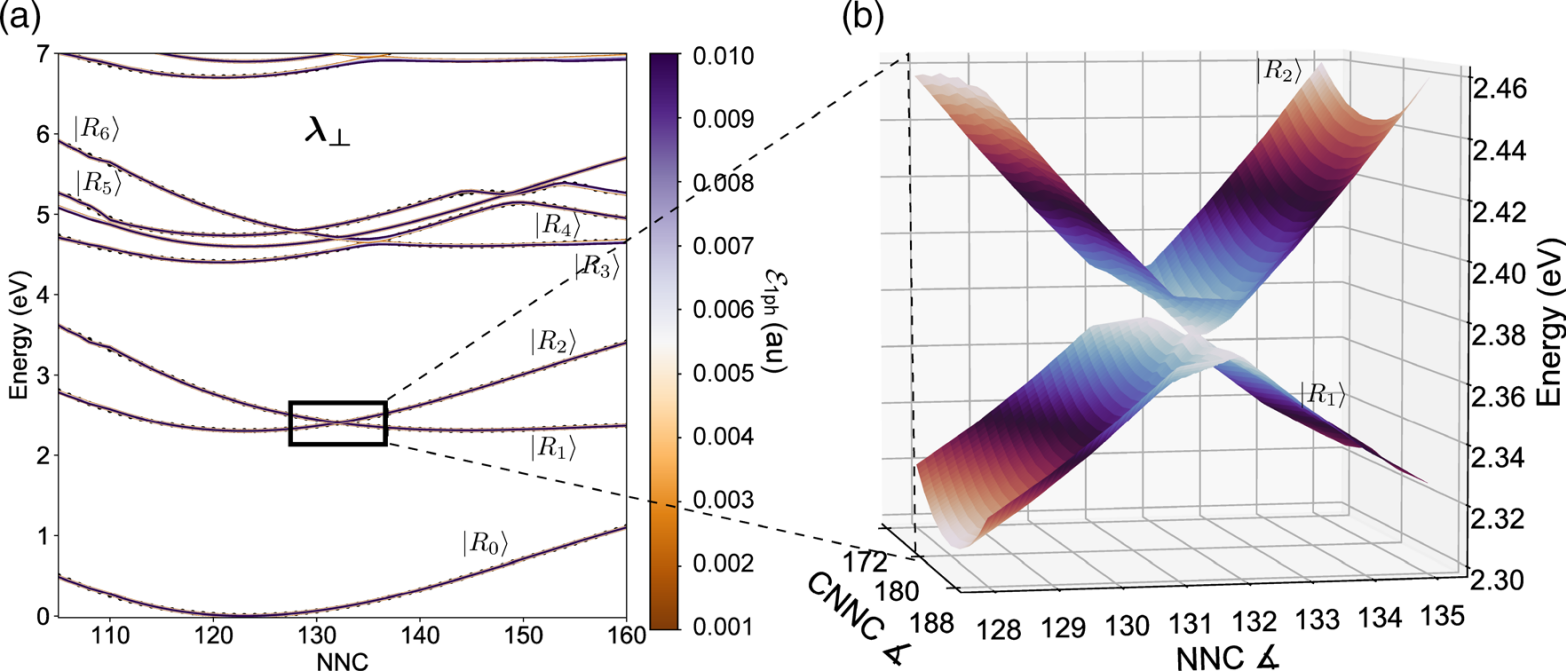
Polaritonic conical intersection for weak to ultrastrong values of ℰ_1*ph*_, with the field polarization perpendicular to the longitudinal axis of the molecule. (a) Polaritonic states computed along the NNC coordinate in the same conditions as in Figure 1a. The polarization of the field ***λ***_⊥_ is perpendicular to the longitudinal axis of the molecule. Along this direction, the vanishing *S*_0_ → *S*_1_ transition dipole moment at azobenzene trans‐planar geometries (CNNC∼175 − 180°) causes (b) a polaritonic conical intersection to arise, independently of the coupling strength [Color figure can be viewed at wileyonlinelibrary.com]

The polarization of the field is another important issue to deal with when computing polaritonic states. Indeed, the anisotropy of the transition dipole moment components with respect to the axes of the molecule impacts the outcoming energy landscape as well. For *trans*‐azobenzene at nearly planar geometries, the largest component of the transition dipole moments lies in the molecular plane. In particular, at *C*_2*h*_ geometries the $〈S_{0}\left|\hat{\mu }\right|S_{1}〉$ transition dipole vanishes. As a consequence, by changing the polarization of the field from longitudinal (***λ***_∥_) to perpendicular (***λ***_⊥_) to the plane of the molecule, the PoPESs change from the ones in Figure 1a to the ones in Figure 2a. In the latter case, the dependence of the splitting on the coupling strength is lost due to a vanishing transition dipole moment when CNNC is 180°.

Although the uncoupled states used in the calculation are the same as in Figure 1, almost all the lines corresponding to different ℰ_1*ph*_ are overlapped in Figure 2a. The transition dipole moments perpendicular to the plane of the molecule begin to rapidly grow when twisting the molecule, that is, with a change along the CNNC coordinate. Consequently, the polaritons are split again by a twisting of the CNNC dihedral, resulting in a polaritonic conical intersection (Figure 2b) at CNNC 180° and NNC 132°. All these features provide a clear evidence that the molecular complexity must be dealt with to correctly describe the photochemical dynamics on polaritonic states.

### 2.4 Analytical gradients for CI‐expanded polaritonic states

After showing the strong coupling contribution to the energy in the previous section (Equation (24)), here we derive the analytical energy gradient with respect to the nuclear coordinates *R*_*α*_ for a generic FOMO‐CI expanded polaritonic state. The present approach is based on previous works,^[^ ^43^, ^58^ ^]^ where the Z‐vector method has been applied. In particular, here we adapt to the polaritonic case the “contracted” strategy that was developed in a spin‐orbit framework.^[^ ^44^ ^]^ As in References [44, 58], only the active MOs are allowed to have floating occupation numbers. The gradient of the energy can be partitioned in a response term, containing the derivatives of CI and MO coefficients, and a static term. The static contribution specific to the present case is given by the derivative of the molecular dipole operator matrix elements in terms of atomic orbitals (AO). As for the response terms, notice that the derivatives of the expansion coefficients $D_{n,p}^{A}$ of the polaritonic adiabatic state ∣*A*〉 give a null contribution to $\frac{\partial E_{\mathrm{pol}}^{A}}{\partial R_{\alpha }}$, since $\frac{\partial E_{\mathrm{pol}}^{A}}{\partial D_{n,p}^{A}}=0$ by construction. As a consequence, since $E_{\mathrm{ph}}^{A}$ does not involve geometry dependent quantities other than the *D*_*A*_ coefficients (in the long wavelength approximation), it does not contribute to the gradient and will not be considered further here. At variance, the derivatives of the electronic CI coefficients *C*_*I*, *n*_ have to be considered.

We have then

(27) $$\frac{\partial E_{\mathrm{pol}}^{A}}{\partial R_{\alpha }}=\frac{\partial E_{\mathrm{el}}^{A}}{\partial R_{\alpha }}+\frac{\partial E_{\mathrm{int}}^{A}}{\partial R_{\alpha }}.$$

The gradients for the electronic energies *U*_*n*_ entering $\frac{\partial E_{\mathrm{el}}^{A}}{\partial R_{\alpha }}$ are known.^[^ ^43^, ^44^, ^46^, ^58^ ^]^ Hence, here we only show explicitly the evaluation of $\frac{\partial E_{\mathrm{int}}^{A}}{\partial R_{\alpha }}$. By making use of Equation (24) one gets

(28) $$\frac{\partial E_{\mathrm{int}}^{A}}{\partial R_{\alpha }}={ℰ}_{1\mathrm{ph}}\sum_{\mathrm{ij}}\left[\frac{\partial \rho_{\mathrm{ij}}^{\mathrm{int}}\left(A\right)}{\partial R_{\alpha }}\mu_{\mathrm{ij}}+\rho_{\mathrm{ij}}^{\mathrm{int}}\left(A\right)\frac{\partial \mu_{\mathrm{ij}}}{\partial R_{\alpha }}\right].$$

Let *μ*_*ij*_ be the matrix element of the molecular dipole operator ${\hat{\mu }}_{\lambda }$ in the MO basis, and **c** the transformation matrix from the AO to the MO set (**c** is real orthogonal in the semiempirical framework considered here). We have then ***μ*** = **c**^†^***μ***_*AO*_**c**, where ***μ***_*AO*_ is the matrix of ${\hat{\mu }}_{\lambda }$ in the AO basis. Therefore, the derivatives of ***μ*** can be expressed as

(29) $$\frac{\partial \mu }{\partial R_{\alpha }}=B^{\alpha }\mu -{\mathrm{\mu B}}^{\alpha }+c^{†}\frac{\partial \mu_{\mathrm{AO}}}{\partial R_{\alpha }}c\;\text{with}\;B^{\alpha }=\frac{\partial c^{†}}{\partial R_{\alpha }}c,$$

which can be decomposed into a static part and a response part,^[^ ^43^, ^44^, ^58^ ^]^

(30) $${\left.\frac{\partial \mu }{\partial R_{\alpha }}\right|}_{\text{static}}=c^{†}\frac{\partial \mu_{\mathrm{AO}}}{\partial R_{\alpha }}c,$$

(31) $${\left.\frac{\partial \mu }{\partial R_{\alpha }}\right|}_{\text{resp}}=B^{\alpha }\mu -{\mathrm{\mu B}}^{\alpha }$$

The static term (Equation (30)) is easily evaluated as follows. Let $\chi_{\sigma }^{\alpha }\left(\vec{R}\right)$ be an AO belonging to nucleus *α* centered on ${\vec{R}}_{\alpha }$, with ${\vec{r}}_{\mathrm{rel}}=\vec{r}-{\vec{R}}_{\alpha }$. The dipole matrix elements *μ*_*στ*_ are then, in the semiempirical framework

(32) $${\vec{\mu }}_{\sigma \tau }=-e〈\chi_{\sigma }^{\alpha }\left({\vec{r}}_{\mathrm{rel}}\right)\left|\vec{r}\right|\chi_{\tau }^{\beta }\left({\vec{r}}_{\mathrm{rel}}\right)〉=-e\delta_{\alpha \beta }\left(\delta_{\sigma \tau }{\vec{R}}_{\alpha }+{\vec{f}}_{\sigma \tau }\right)$$

where −*e* is the electronic charge and the Kronecker delta *δ*_*αβ*_ is due to the semiempirical NDDO approximation. Moreover, the term

(33) $${\vec{f}}_{\sigma \tau }=〈\chi_{\sigma }^{\alpha }\left({\vec{r}}_{\mathrm{rel}}\right)\left|{\vec{r}}_{\mathrm{rel}}\right|\chi_{\tau }^{\beta }\left({\vec{r}}_{\mathrm{rel}}\right)〉$$

is independent on the nuclear coordinates. Therefore, the derivative of ${\vec{\mu }}_{\sigma \tau }$ with respect to ${\vec{R}}_{\alpha }$ vanishes unless the two atomic orbitals *σ* and *τ* are both centered on the nucleus *α*, and in that case it simply evaluates to $-e\frac{\partial {\vec{R}}_{\alpha }}{\partial {\vec{R}}_{\alpha }}$. In an ab initio context, one would have to compute also the derivative of the dipole matrix elements between atomic orbitals centered on different atoms, which has a more involved expression with respect to the term considered here. However, that would not be expected to have a large impact on the computational cost, which is mainly influenced by the response part of the gradient.

The contribution of the response term of Equation (31) to the derivative of $E_{\mathrm{int}}^{A}$ (Equation (28)) can be recast in this way, following Patchkovskii and Thiel^[^ ^45^, ^46^ ^]^

(34) $${\left.{ℰ}_{1\mathrm{ph}}\sum_{\mathrm{ij}}\rho_{\mathrm{ij}}^{\mathrm{int}}\left(A\right)\frac{\partial \mu_{\mathrm{ij}}}{\partial R_{\alpha }}\right|}_{\text{resp}}=\sum_{i}\sum_{a}\left(B_{\mathrm{ia}}^{\alpha }+\frac{\partial \varepsilon_{i}}{\partial R_{\alpha }}\delta_{\mathrm{ia}}\right)q_{\mathrm{ia}}^{\mathrm{int}},$$

where *ε*_*i*_ is the energy of MO *i* and

(35) $$\begin{array}{ll} q_{\mathrm{ii}}^{\mathrm{int}} & =0\\ q_{\mathrm{ia}}^{\mathrm{int}} & =2{ℰ}_{1\mathrm{ph}}\sum_{j}\rho_{\mathrm{ij}}^{\mathrm{int}}\left(A\right)\mu_{\mathrm{aj}}\;i\neq a. \end{array}$$

As $q_{\mathrm{ii}}^{\mathrm{int}}=0$, the term containing the derivative of the orbital energy *ε*_*i*_ gives a null contribution to the sum of Equation (34). Such term has been included to recover the same formalism of previous works.^[^ ^43^, ^44^, ^58^ ^]^

We now turn to the derivative of $\rho_{\mathrm{ij}}^{\mathrm{int}}\left(A\right)$, which is a response term (i.e., the CI response contribution to the polaritonic energy), evaluated by taking the derivative of $\Delta_{\mathrm{ij}}^{\mathrm{el}}\left(m,n\right)$. Such derivative is obtained by following the same procedure outlined for the MOs response terms (Equation (31))

(36) $$\frac{\partial \Delta_{\mathrm{ij}}^{\mathrm{el}}\left(m,n\right)}{\partial R_{\alpha }}=\sum_{k}^{N_{\mathrm{CI}}}\left(d_{\mathrm{mk}}^{\alpha }\Delta_{\mathrm{ij}}^{\mathrm{el}}\left(k,n\right)-\Delta_{\mathrm{ij}}^{\mathrm{el}}\left(m,k\right)d_{\mathrm{kn}}^{\alpha }\right)$$

with

(37) $$d_{\mathrm{mn}}^{\alpha }=\sum_{J}\frac{\partial C_{\mathrm{Jm}}}{\partial R_{\alpha }}C_{\mathrm{Jn}}$$

Notice that the sum in Equation (36) is extended to *N*_*CI*_ rather than to the number *N* of states selected: in principle, the evaluation of the CI response contribution requires the full diagonalization of the CI space considered. While this may be too demanding in an ab initio context, normally it does not represent a problem in a semiempirical framework, where *N*_*CI*_ is usually small. The antisymmetric matrix $d_{\mathrm{nm}}^{\alpha }$, expressing the response of the CI coefficients, represents the CI contribution to the derivative couplings. We have then

$${\left.\frac{\partial E_{\mathrm{int}}^{A}}{\partial R_{\alpha }}\right|}_{\text{resp}}^{\mathrm{CI}}\equiv {ℰ}_{1\mathrm{ph}}\sum_{\mathrm{ij}}\frac{\partial \rho_{\mathrm{ij}}^{\mathrm{int}}\left(A\right)}{\partial R_{\alpha }}\mu_{\mathrm{ij}}$$

(38) $$=\sum_{i\leq j}\sum_{n\neq m}G_{\mathrm{ij}}\left(A|m,n\right)\sum_{k}^{N_{\mathrm{CI}}}\left(d_{\mathrm{mk}}^{\alpha }\Delta_{\mathrm{ij}}^{\mathrm{el}}\left(k,n\right)+d_{\mathrm{nk}}^{\alpha }\Delta_{\mathrm{ij}}^{\mathrm{el}}\left(m,k\right)\right),$$

where

(39) $$G_{\mathrm{ij}}\left(A|m,n\right)={ℰ}_{1\mathrm{ph}}D\left(A|m,n\right)\mu_{\mathrm{ij}}\left(2-\delta_{\mathrm{ij}}\right).$$

According to Reference [44], we evaluate the derivative coupling terms $d_{\mathrm{mn}}^{\alpha }$ by exploiting the Hellmann–Feynman theorem

(40) $$d_{\mathrm{mn}}^{\alpha }={\left(U_{m}-U_{n}\right)}^{-1}\sum_{\mathrm{IJ}}C_{I,m}\frac{\partial 〈\Phi_{I}|{\hat{H}}_{\mathrm{el}}|\Phi_{J}〉}{\partial R_{\alpha }}C_{J,n}$$

(41) $$=\sum_{\mathrm{ij}}\frac{\Delta_{\mathrm{ij}}^{\mathrm{el}}\left(m,n\right)}{U_{m}-U_{n}}\frac{\partial \varepsilon_{\mathrm{ij}}^{+}}{\partial R_{\alpha }}+\sum_{\text{ijkl}}\frac{\Gamma_{\text{ijkl}}^{\mathrm{el}}\left(m,n\right)}{U_{m}-U_{n}}\frac{\partial 〈\mathrm{ij}|\mathrm{kl}〉}{\partial R_{\alpha }}.$$

for *m* ≠ *n*, and $d_{\mathrm{nn}}^{\alpha }=0$. Here, $\Gamma_{\text{ijkl}}^{\mathrm{el}}\left(m,n\right)=〈m\left|{\hat{a}}_{i}^{†}{\hat{a}}_{j}^{†}{\hat{a}}_{l}{\hat{a}}_{k}\right|n〉$ are the two‐electron density matrices and the terms $\varepsilon_{\mathrm{ij}}^{+}$ are defined in equation (36) of Reference [44].

Inserting Equation (41) into (38), we obtain the following expression for the CI response term induced by the strong coupling interaction:

(42) $${\left.\frac{\partial E_{\mathrm{int}}^{A}}{\partial R_{\alpha }}\right|}_{\text{resp}}^{\mathrm{CI}}=\sum_{\mathrm{ij}}\frac{\partial \varepsilon_{\mathrm{ij}}^{+}}{\partial R_{\alpha }}\Delta_{\mathrm{ij}}^{\mathrm{int}}\left(A\right)+\sum_{\text{ijkl}}\frac{\partial 〈\mathrm{ij}|\mathrm{kl}〉}{\partial R_{\alpha }}\Gamma_{\text{ijkl}}^{\mathrm{int}}\left(A\right).$$

where

(43) $$\Delta_{\mathrm{ij}}^{\mathrm{int}}\left(A\right)=\sum_{k}^{N_{\mathrm{CI}}}\sum_{\begin{array}{c} m\\ m\neq k \end{array}}\frac{\Delta_{\mathrm{ij}}^{\mathrm{el}}\left(m,k\right)}{U_{m}-U_{k}}R\left(A|k,m\right)$$

(44) $$\Gamma_{\text{ijkl}}^{\mathrm{int}}\left(A\right)=\sum_{k}^{N_{\mathrm{CI}}}\sum_{\begin{array}{c} m\\ m\neq k \end{array}}\frac{\Gamma_{\text{ijkl}}^{\mathrm{el}}\left(m,k\right)}{U_{m}-U_{k}}R\left(A|k,m\right)$$

(45) $$R\left(A|k,m\right)=\sum_{i\leq j}\sum_{\begin{array}{c} n\\ n\neq m \end{array}}2G_{\mathrm{ij}}\left(A|m,n\right)\Delta_{\mathrm{ij}}^{\text{symm}}\left(k,n\right)$$

(46) $$\Delta_{\mathrm{ij}}^{\text{symm}}\left(k,n\right)=\frac{\Delta_{\mathrm{ij}}^{\mathrm{el}}\left(k,n\right)+\Delta_{\mathrm{ji}}^{\mathrm{el}}\left(k,n\right)}{2}$$

Here, $\Delta_{\mathrm{ij}}^{\text{symm}}\left(k,n\right)$ is the symmetric part of $\Delta_{\mathrm{ij}}^{\mathrm{el}}\left(k,n\right)$. Notice that it is symmetric with respect to both *i*, *j* and *k*, *n* indices, since $\Delta_{\mathrm{ij}}^{\mathrm{el}}\left(k,n\right)=\Delta_{\mathrm{ji}}^{\mathrm{el}}\left(n,k\right)$.

To obtain the final expression for the gradient of $E_{\mathrm{pol}}^{A}$, we have also to consider the contribution given by the derivative of the naked electronic state energy *U*_*n*_ (see Reference [58])

(47) $$\frac{\partial U_{n}}{\partial R_{\alpha }}=\frac{\partial E_{0}}{\partial R_{\alpha }}+\sum_{\mathrm{ij}}\Delta_{\mathrm{ij}}^{\mathrm{el}}\left(n\right)\frac{\partial \varepsilon_{\mathrm{ij}}^{+}}{\partial R_{\alpha }}+\sum_{\text{ijkl}}\Gamma_{\text{ijkl}}^{\mathrm{el}}\frac{\partial 〈\mathrm{ij}|\mathrm{kl}〉}{\partial R_{\alpha }}.$$

By putting all the terms together we arrive at

(48) $$\begin{array}{ll} \frac{\partial E_{\mathrm{pol}}^{A}}{\partial R_{\alpha }}= & \frac{\partial E_{0}}{\partial R_{\alpha }}+\sum_{\mathrm{ij}}\Delta_{\mathrm{ij}}^{\mathrm{pol}}\left(A\right)\frac{\partial \varepsilon_{\mathrm{ij}}^{+}}{\partial R_{\alpha }}+\sum_{\text{ijkl}}\Gamma_{\text{ijkl}}^{\mathrm{pol}}\left(A\right)\frac{\partial 〈\mathrm{ij}|\mathrm{kl}〉}{\partial R_{\alpha }}\\ & +\sum_{\mathrm{ai}}B_{\mathrm{ia}}^{\alpha }q_{\mathrm{ia}}^{\mathrm{int}}+{ℰ}_{1\mathrm{ph}}\sum_{\mathrm{ij}}\rho_{\mathrm{ij}}^{\mathrm{int}}\left(A\right)\sum_{\sigma \tau }c_{\mathrm{\sigma i}}\frac{\partial \mu_{\sigma \tau }}{\partial R_{\alpha }}c_{\mathrm{\tau j}} \end{array}$$

where we made use of the modified electronic density matrices

(49) $$\Delta_{\mathrm{ij}}^{\mathrm{pol}}\left(A\right)=\sum_{n}\Delta_{\mathrm{ij}}^{\mathrm{el}}\left(n\right)\sum_{p}{\left|D_{n,p}^{A}\right|}^{2}+\Delta_{\mathrm{ij}}^{\mathrm{int}}\left(A\right)$$

(50) $$\Gamma_{\text{ijkl}}^{\mathrm{pol}}\left(A\right)=\sum_{\text{ijkl}}\Gamma_{\text{ijkl}}^{\mathrm{el}}\left(n\right)\sum_{p}{\left|D_{n,p}^{A}\right|}^{2}+\Gamma_{\text{ijkl}}^{\mathrm{int}}\left(A\right).$$

The evaluation of the gradient of $E_{\mathrm{pol}}^{A}$ can proceed in the way outlined in Reference [58], using the modified density matrices $\Delta_{\mathrm{ij}}^{\mathrm{pol}}\left(A\right)$ and $\Gamma_{\text{ijkl}}^{\mathrm{pol}}\left(A\right)$. In particular, the response term is

(51) $${\left.\frac{\partial E_{\mathrm{pol}}^{A}}{\partial R_{\alpha }}\right|}_{\text{resp}}=\sum_{i}\sum_{a}\left(B_{\mathrm{ia}}^{\alpha }+\frac{\partial \varepsilon_{i}}{\partial R_{\alpha }}\right)\left(q_{\mathrm{ia}}^{\mathrm{el}}+q_{\mathrm{ia}}^{\mathrm{int}}\right),$$

where *q*^*el*^, defined as done in^[^ ^58^ ^]^ (see also^[^ ^44^ ^]^), is explicitly reported below for reader's convenience

(52) $$q_{\mathrm{ii}}^{\mathrm{el}}=\Delta_{\mathrm{ii}}^{\mathrm{pol}}\left(A\right)-O_{i}-\frac{1}{2}\sum_{\mathrm{jkl}}\beta_{\mathrm{ki}}〈\mathrm{lj}\|\mathrm{kk}〉\left(\Delta_{\mathrm{lj}}^{\mathrm{pol}}\left(A\right)-\delta_{\mathrm{lj}}O_{l}\right)$$

(53) $$q_{\mathrm{ia}}^{\mathrm{el}}=4\sum_{\mathrm{jkl}}\Gamma_{\text{ijkl}}^{\mathrm{pol}}\left(A\right)〈\mathrm{aj}|\mathrm{kl}〉-\sum_{\mathrm{jk}}\Delta_{\mathrm{ij}}^{\mathrm{pol}}\left(A\right)O_{k}〈\mathrm{kk}\|\mathrm{aj}〉-$$

$$\;\sum_{\mathrm{jk}}\Delta_{\mathrm{jk}}^{\mathrm{pol}}\left(A\right)O_{i}〈\mathrm{ai}\|\mathrm{jk}〉+\sum_{j}O_{i}O_{j}〈\mathrm{ai}\|\mathrm{jj}〉\;\left(\text{for}\;i\neq a\right)$$

(54) $$\beta_{\mathrm{ki}}=f_{k}\left(\varepsilon_{F}\right)\left(\frac{f_{i}\left(\varepsilon_{F}\right)}{\sum_{j}f_{j}\left(\varepsilon_{F}\right)}-\delta_{\mathrm{ik}}\right)$$

In the above equations, we used the shorthand 〈*ij*‖*kl*〉 = 2〈*ij*| *kl*〉 − 〈*ik*| *jl*〉, and *f*_*i*_ is the Gaussian function defined in Equation (10). Finally, for the static part, one has just to add the last term of Equation (48), representing the static dipole derivative (see above).

### 2.5 Surface hopping

In the framework of Direct Trajectory Surface Hopping, the formulation of strong coupling given in this work allows to include the decoherence corrections^[^ ^59^ ^]^ and environmental effects through the QM/MM interface previously devised.^[^ ^60^, ^61^, ^62^ ^]^ For the time evolution of the polaritonic wavefunction, we adopt the local diabatization technique,^[^ ^48^, ^63^ ^]^ with a recently improved evaluation of transition probabilities. Such probabilities are compliant with Tully's Fewest Switches prescription and particularly effective when many states are involved in nonadiabatic events,^[^ ^64^ ^]^ as commonly happens in single‐molecule polaritonic systems (see Figure 1b).

**FIGURE 3:**
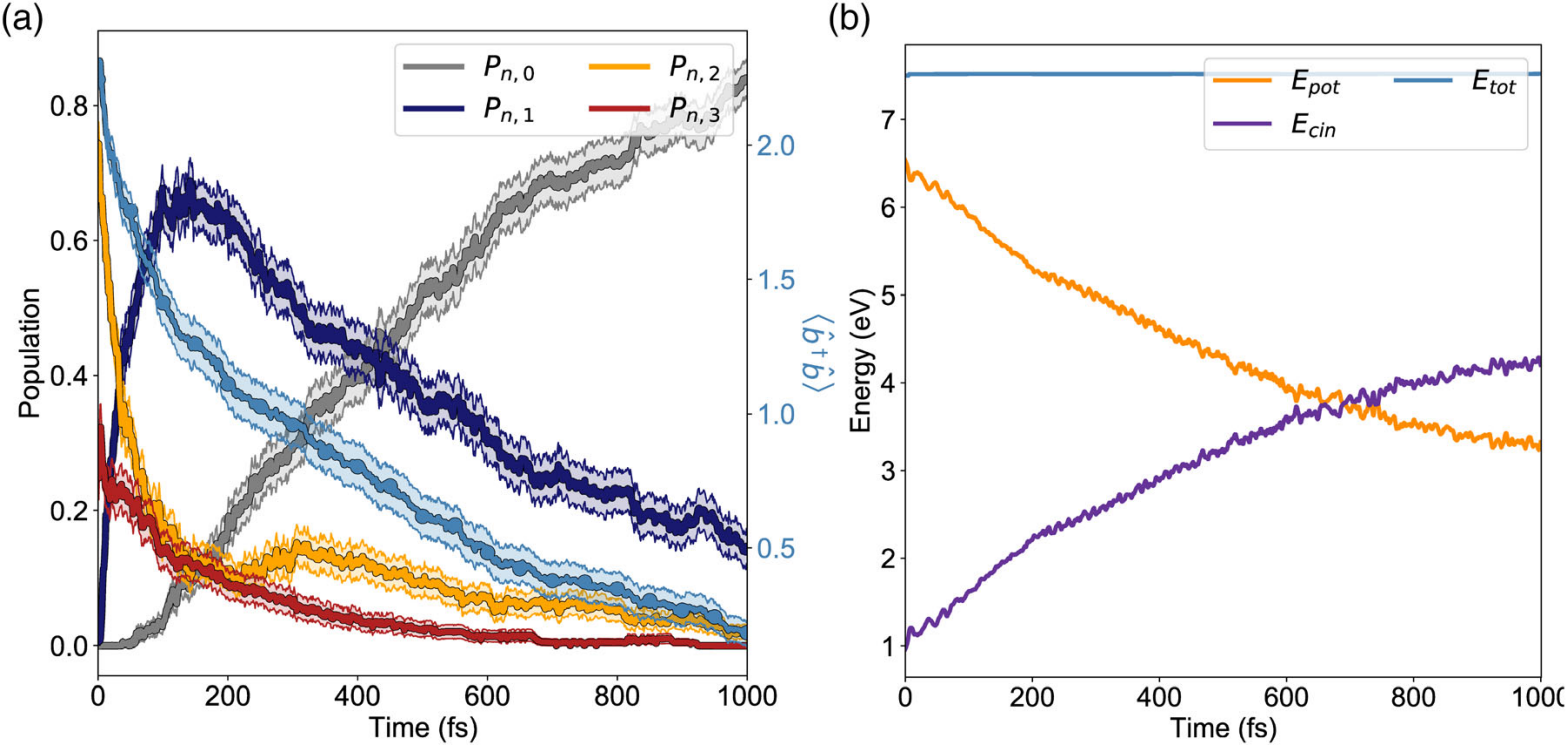
Photon statistics and energy conservation. (a) Dynamics of the photons in the cavity during the internal relaxation of the strongly‐coupled azobenzene molecule, in absence of cavity losses. The dynamics is started from the ∣*R*_8_〉 state and runs for 1 ps, with ℰ_1*ph*_ = 0.004 au (∼12 nm^3^ mode volume), *ω*_*ph*_ = 2.7 eV and longitudinal field polarization. The molecule is in gas phase.^[^ ^24^ ^]^ The curves with full lines show the dynamics of each *p* subspace, while the light blue line with circle markers (with the scale on the right) represents the total photon number within the cavity. Error bars, represented as lighter bands, are also shown. Even in absence of cavity losses, the average photon number decreases during the dynamics. While the photon is in its absorbed state, the energy stored within the molecule is redistributed via internal conversion to nuclear kinetic energy. The overall process is still conserving the energy, as shown in panel (b) [Color figure can be viewed at wileyonlinelibrary.com]

As a test case, we examine the azobenzene strong coupling dynamics with *ω*_*ph*_ = 2.7 eV and ℰ_1*ph*_ = 0.004 au (∼12 nm^3^) in the absence of the cavity losses. The initial conditions are sampled on the ground state via a 20 ps dynamics, thermostated at room temperature (with a Bussi‐Parrinello thermostat^[^ ^65^ ^]^). In particular, 230 starting structures and velocities are extracted from the sampling dynamics, and the system is initially vertically excited to the ∣*R*_8_〉 state, that is mostly ∣*S*_2_, 2〉 state. Rather than the simulation of a realistic excitation (the transition ∣*S*_0_, 0〉 →  ∣ *S*_2_, 2〉 would require a multiphoton pumping), this is a test case to investigate the effect of photon occupation numbers greater than 1 (up to *p* = 3). In Figure 3a, we show the behavior of the population of the photon states during the dynamics, in the absence of cavity losses. The blue line with circle markers (right y scale) shows the total photon number within the cavity, namely $〈{\hat{b}}^{†}\hat{b}〉$.

The full lines (left y scale) show the populations of each photon state, that is, ∑_*n*_|*D*_*n*, *p*_|^2^, with *p* = 0, …, 3. While no cavity loss is explicitly included in the dynamics, still the total photon number in the system decreases. Through strong coupling, a photon is continuously exchanged between the molecule and the cavity. However, the electronic component keeps decaying via internal conversion, meaning that when the photon is absorbed, its energy can be redistributed to the nuclear degrees of freedom. While the total number of photons decreases in the ongoing dynamics, the energy of the system is still conserved (Figure 3b). The initial conditions are chosen such that the resonant region between the *p* = 3 states (namely *S*_0_, 3) and the *p* = 2 states ∣*S*_1_, 2〉 is interested, so both the subspaces ∣*n*, 3〉 and ∣*n*, 2〉 are populated. This results into an average photon occupation number greater than ∣2〉, namely 2.23.

### 2.6 Cavity losses

Aiming to provide a realistic model, we deal with the issue of lossy cavities. The strong coupling regime for single molecules is usually reached by exploiting a nanocavity setup of the system.^[^ ^5^, ^6^, ^66^, ^67^ ^]^ The typical lifetime of nanocavities is few tens of femtoseconds. However, we have recently shown that the overall photon lifetime of the system is way longer than the individual cavity lifetime,^[^ ^34^ ^]^ reaching a time scale comparable to several ultrafast photochemical processes. This effect is due to the transient passage of the wavepacket through strongly coupled regions, so that the composition of the polaritonic state keeps interchanging between electronic and photonic. As a consequence of the mixing, the lifetime of states with the photon partially absorbed is extended up to hundreds of fs, depending on the strong coupling conditions. Within our model, we adapt a quantum jump algorithm^[^ ^68^, ^69^, ^70^, ^71^ ^]^ already exploited in the Stochastic Schrödinger Equation (SSE) framework^[^ ^72^ ^]^ to account for relaxation and dephasing channels. Stochastic methods in the framework of SSE are also commonly exploited as an equivalent alternative to master equations in treating cavity losses.^[^ ^73^, ^74^, ^75^ ^]^ We then follow a standard implementation of this approach, similar to others already present in Quantum Optics simulation packages like QuTip.^[^ ^76^ ^]^

The quantum jump is a natural choice as it fully exploits the trajectory‐based machinery of the surface hopping. Having to deal with semiclassical trajectories, both the polaritonic wavefunction and the “current state” (i.e., the adiabatic state on which PES the nuclei are evolving) must be taken into account whenever a photon loss occurs. We start with the expression of the polaritonic wavefunction in terms of the uncoupled states basis:

(55) $$\Psi_{\mathrm{pol}}=\sum_{A}C_{A}|A〉=\sum_{n,p}d_{n,p}|n,p〉.$$

where $d_{n,p}=\sum_{A}D_{n,p}^{A}C_{A}$. Only states with free photons can decay via cavity losses, namely states with *p* ≥ 1. We evaluate the photon loss probability *P*_*dec*_ by taking the squared modulus of the uncoupled states coefficients with *p* ≥ 1 in the total wavefunction Ψ_*pol*_, that is, *d*_*n*, *p* ≥ 1_:

(56) $$P_{\mathrm{dec}}=\sum_{p\geq 1,n}{\left|d_{n,p}\right|}^{2}\frac{\Delta t}{\tau }.$$

Here, Δ*t* is the integration time step and *τ* is the cavity lifetime, namely the inverse of the cavity decay rate *κ*. We generate a uniform random number within the interval [0, 1]. If the random number falls in [*P*_*dec*_, 1], the photon is retained and the cavity loss does not occur. If not, the photon is lost. Upon photon loss, the photon occupation number is lowered by 1 via application of the projector $\hat{P}$ which includes the photon annihilation operator $\hat{b}$:

(57) $$\hat{P}\Psi_{\mathrm{pol}}=\left(I_{\mathrm{el}}\;⊗\;\hat{b}\right)\Psi_{\mathrm{pol}}=\sum_{n}\sum_{p=1}^{p_{\max}}d_{n,p-1}\sqrt{p}|n,p-1〉.$$

Here, the projector $\hat{P}$ preserves the electronic coherence within each p subspace, apart of course for *p* = 0 that is annihilated. The photonic annihilation operator $\hat{b}$ is applied to mimic the loss of the photon from the cavity, resulting in a manifold of states with photon number lowered by one. The wavefunction is normalized after application of P.

To reinitialize the dynamics after the photon loss has occurred, we need both the wavefunction to propagate and a polaritonic surface to resume the nuclear trajectory integration, ∣*F*〉. The wavefunction is simply a linear combination of polaritonic states ∣*A*〉:

(58) $$\Psi_{\mathrm{pol}}^{\prime }=\hat{P}\Psi_{\mathrm{pol}}=\sum_{A}C_{A}^{\prime }|A〉,$$

where the ' symbol denotes the quantities after the jump. As a polaritonic energy surface to resume the nuclear trajectory propagation, we choose the polaritonic surface ∣*F*〉 that has the maximum overlap with the polaritonic wavefunction after the jump:

(59) $$|F〉=|A〉\;|\;\max\left\{\left|<{\mathrm{A|\Psi}}_{pol}^{\prime }>\right|\right\}.$$

If the quantum jump does not occur, the wavefunction is propagated with the non‐Hermitian Hamiltonian^[^ ^68^, ^72^, ^77^ ^]^:

(60) $${\hat{H}}_{\mathrm{eff}}={\hat{H}}_{\mathrm{pol}}-i\hbar \frac{\kappa }{2}{\hat{b}}^{†}\hat{b}.$$

For each timestep, this is accomplished by first propagating according to *H*_*pol*_, and then modifying Ψ_*pol*_ in the following way

(61) $$\Psi_{\mathrm{pol}}=\sum_{A}\sum_{n,p}C_{A}D_{n,p}^{A}\left(1-\frac{\kappa }{2}p\Delta t\right)|n,p〉.$$

The polaritonic wavefunction Ψ_*pol*_ is then renormalized. The propagation between each attempted jump should be performed with the non‐Hermitian ${\hat{H}}_{\mathrm{eff}}$ of Equation (60), leading to un‐normalized wavefunction. Anyway, in our algorithm, the jump is attempted at each time step and so the wavefunction is always normalized, one way or the other. A consequence of the photon loss is that the total energy of the system is not conserved.

**FIGURE 4:**
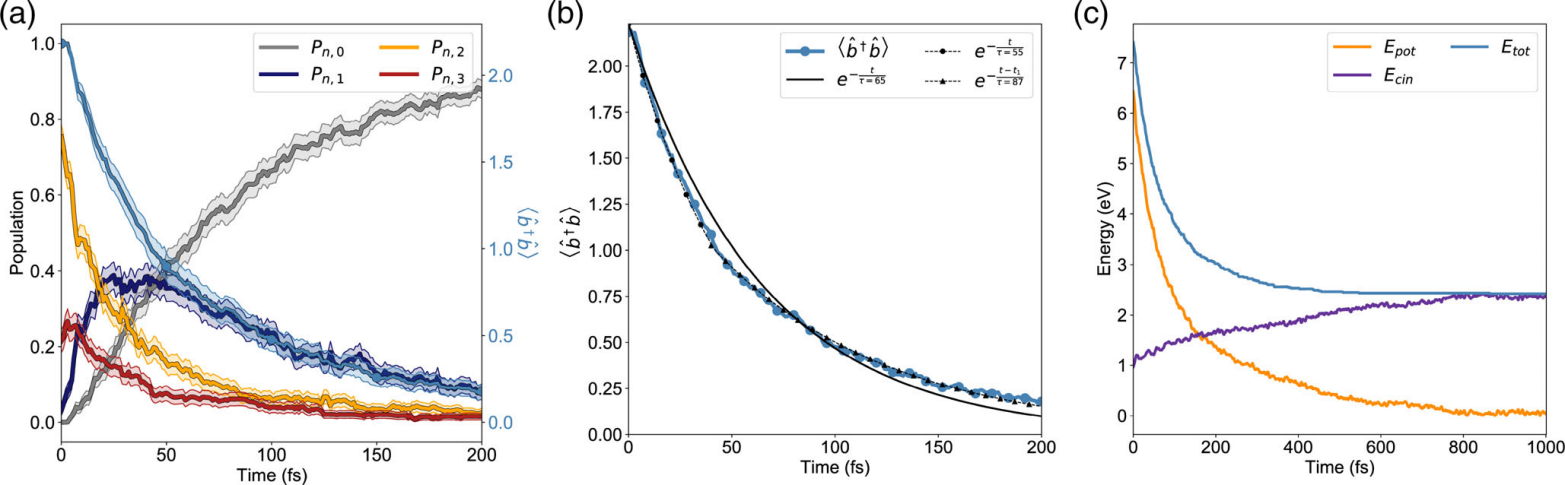
Cavity losses in strong coupling. Same conditions of Figure 3, with the same notation and color scheme. A cavity lifetime *τ* = 65 fs is considered. (a) The overall population dynamics is definitely shorter in this case, with a transient population of the ∣*n*, 1〉 subspace. (b) Photon number in the cavity at each time step. Remarkably, the kinetics is not simply dissipative. While *p* ≥ 1, the photon loss occurs at a faster rate than the cavity lifetime (circle markers fit). After only one photon remains, the loss dynamics slows down, as the only photon remaining is partially absorbed by the molecule and cannot be lost. (c) Breakdown of the energy conservation, due to cavity losses [Color figure can be viewed at wileyonlinelibrary.com]

In Figure 4, we replicate the dynamics performed for the lossless case (Figure 3) in presence of a cavity lifetime *τ* of 65 fs. The same color scheme and notation is applied. While the relaxation dynamics is of course quicker (Figure 4a) due to the presence of an extra relaxation channel (cavity loss), the decay dynamics is better described by a biexponential function, rather than a simple exponential (Figure 4b).

The main reason is that photons can be exchanged back and forth between the cavity and the molecule, via transitions ∣*n*, *p*〉 →  ∣ *n* + 1, *p* − 1〉 and vice versa, slowing down the cavity loss rate. This is especially important when *p* = 1, as there is no way to lose the photon from a state ∣*n*^′^, 0〉 with zero free photons in the cavity. In particular, this happens for the system considered here, which shows transitions back and forth from ∣*S*_1_, 0〉 to ∣*S*_0_, 1〉. Here, the single photon remaining appears to decay with a lifetime which is 20 fs longer than the nominal decay time of the cavity. Notice that, if the single photon remaining is adsorbed by the molecule due to strong coupling, the lifetime of the system is ascribable to that of the pure electronic states. Conversely, when the photon is free within the cavity, the lifetime of the system becomes that of the nominal cavity lifetime.

The consequence of the cavity losses becomes also evident in the energy conservation plot (Figure 4c), where the initial part of the dynamics is characterized by a quick drop of the total energy due to the photon losses with no kinetic energy compensation. As a last remark, we stress that the current implementation takes advantage of dressing the chemical quantities for the strong coupling effect. Consequently, it directly supports the interface with the TINKER package to perform QM/MM simulations with electrostatic embedding, as described and applied in References [34, 61, 62].

## 3 CONCLUSIONS

In the present work, we describe a scheme we have implemented to perform direct nonadiabatic molecular dynamics simulations for semiclassical molecules in strong coupling, based on classical nuclear trajectories and on multiconfigurational wavefunctions. We build polaritonic states and present the evaluation of analytical gradients for polaritonic CI energies, extending the DTSH machinery to the polaritonic systems. Among the DTSH^[^ ^42^, ^43^, ^58^ ^]^ exploitable features, we count the decoherence corrections,^[^ ^59^ ^]^ the QM/MM interface with electrostatic embedding^[^ ^60^, ^62^ ^]^ and the local diabatization scheme^[^ ^48^, ^63^ ^]^ for wavefunction propagation. Cavity losses are included in the simulations through quantum jumps, relying on the stochastic nature of Surface Hopping. We choose the test case to highlight the complex features of the potential energy surfaces arising when moving beyond the one‐dimensional 2‐level molecular models. The results presented for the test dynamics highlight the delicate interplay between radiative and nonradiative emissions, both impacting the relaxation dynamics of strongly coupled systems. Especially, we show that losses are competitive with usual nonadiabatic events and that the outcoming dynamics cannot be described as simply dissipative, the photon actually living longer than the nominal lifetime of the cavity. The content of this work provides both formal and conceptual tools to approach the polaritonic photochemical simulations within a semiclassical ansatz, allowing to simulate complete photochemical reactions with a trivially parallelizable technique.
